# Overview of how N32 and N34 elovanoids sustain sight by protecting retinal pigment epithelial cells and photoreceptors

**DOI:** 10.1194/jlr.TR120001137

**Published:** 2021-03-02

**Authors:** Nicolas G. Bazan

**Affiliations:** Neuroscience Center of Excellence, School of Medicine, Louisiana State University Health Sciences Center, New Orleans, LA, USA

**Keywords:** adiponectin receptor 1, age-related macular degeneration, C1q tumor necrosis factor-related protein-5, elovanoids, MALDI IMS, membrane-type frizzled-related protein, neuroprotectin D1, interphotoreceptor matrix, very long-chain polyunsaturated fatty acids, Senescence gene programming, AD, Alzheimer's disease, AdipoR1, adiponectin receptor 1, AMD, age-related macular degeneration, ELOVL4, Elongation of very long-chain fatty acids-4, ELV, elovanoid, ERG, electroretinogram, IPM, interphotoreceptor matrix, MFRP, membrane-type frizzled-related protein, NPD1, neuroprotectin D1, OAβ, oligomeric amyloid-β, PC, phosphatidylcholine, PLA1, phospholipase A1, PRC, photoreceptor cell, RPE, retinal pigment epithelium, SASP, senescence-associated secretory phenotype, UOS, uncompensated oxidative stress, VLC-PUFA, very long-chain PUFA,n-3

## Abstract

The essential fatty acid DHA (22:6, omega-3 or n-3) is enriched in and required for the membrane biogenesis and function of photoreceptor cells (PRCs), synapses, mitochondria, etc. of the CNS. PRC DHA becomes an acyl chain at the sn-2 of phosphatidylcholine, amounting to more than 50% of the PRC outer segment phospholipids, where phototransduction takes place. Very long chain PUFAs (n-3, ≥ 28 carbons) are at the sn-1 of this phosphatidylcholine molecular species and interact with rhodopsin. PRC shed their tips (DHA-rich membrane disks) daily, which in turn are phagocytized by the retinal pigment epithelium (RPE), where DHA is recycled back to PRC inner segments to be used for the biogenesis of new photoreceptor membranes. Here, we review the structures and stereochemistry of novel elovanoid (ELV)-N32 and ELV-N34 to be ELV-N32: (14*Z*,17*Z*,20*R*,21*E*,23*E*,25*Z*,27*S*,29*Z*)-20,27-dihydroxydo-triaconta-14,17,21,23,25,29-hexaenoic acid; ELV-N34: (16*Z*,19*Z*,22*R*,23*E*,25*E*,27*Z*,29*S*,31*Z*)-22,29-dihydroxytetra-triaconta-16,19,23,25,27,31-hexaenoic acid. ELVs are low-abundance, high-potency, protective mediators. Their bioactivity includes enhancing of antiapoptotic and prosurvival protein expression with concomitant downregulation of proapoptotic proteins when RPE is confronted with uncompensated oxidative stress. ELVs also target PRC/RPE senescence gene programming, the senescence secretory phenotype in the interphotoreceptor matrix, as well as inflammaging (chronic, sterile, low-grade inflammation). An important lesson on neuroprotection is highlighted by the ELV mediators that target the terminally differentiated PRC and RPE, sustaining a beautifully synchronized renewal process. The role of ELVs in PRC and RPE viability and function uncovers insights on disease mechanisms and the development of therapeutics for age-related macular degeneration, Alzheimer's disease, and other pathologies.

The interactions between membrane lipids and proteins are central to most aspects of contemporary biology. The exploration of fundamental questions regarding how lipids, particularly those of the n-3 family of FAs, participate in cell function leads us to identify mediators and signaling that sustain homeostasis and regulate functional cell integrity. At the same time, this approach allows us to interrogate disease models concerning the significance of mediators that support cell survival at the early stages of pathologies. The onset and progression of vision loss prompt complex events that unsettle homeostasis, including inflammatory responses that attempt to restore cell integrity by removing or limiting the effects of triggering factors. These encompass autocrine and/or paracrine chemokines, cytokines, and protective lipid mediators (1, 2, 3, 4). Engaged cells consist of immune cells, blood vessel cells, neurons, Mueller cells, retinal pigment epithelium (RPE) cells, and more with a collective objective of sustaining homeostasis by removing the triggering factors and cell debris toward cell restoration. Here, we review prohomeostatic signaling signatures elicited by elovanoids (ELVs), which modulate inflammatory responses and neuroprotection (Fig. 1). This includes senescence gene programming as well as inflammaging, a form of low-grade, sterile, chronic inflammatory response that we examine here as counteracted by ELVs. The term inflammaging or inflamm-aging derives from “inflammation” and “aging,” because it was often related to senescence. However, it is now recognized that both senescence programming and inflammaging occur at all stages of life span, including early development, cell differentiation, and in many pathological conditions.

**Fig. 1:**
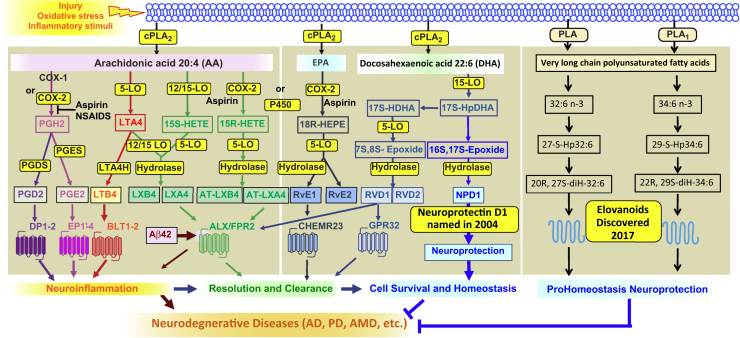
Eicosanoids, docosanoids, and elovanoids. PLAs that release AA, EPA, DHA, or VLC-PUFAs,n-3 are depicted at the top. Synthesis of mediators and receptors involved are illustrated. The outcome is modulation of inflammatory responses and homeostasis. AD, Alzheimer's disease; AMD, age-related macular degeneration; VLC-PUFA, very long-chain PUFA.

A fundamental question concerning sight during aging and in retinal degenerative diseases is how the selective acquisition and retention of key retinal lipidome molecules occur and how these lipids sustain transcriptomic programs and functional integrity. To counter homeostatic disruptions and the onset of neurodegenerations, neuroprotective pathways that control procell and anticell survival signaling are activated. Neurotrophins are a good example of such strategic controlling mechanisms. Also, the recently uncovered low-abundance, high-potency lipid mediators, the ELVs, are important for the cellular and functional integrity of the CNS, including the retina.

## DHA esterified into phospholipids is most abundant in photoreceptors and synaptic membranes

DHA (22:6, omega-3 or n-3) is an essential FA family member highly concentrated in and required for the biogenesis of synaptic membranes and other membranes of the CNS, including photoreceptor cell (PRC) membranes (5). Dietary DHA is supplied or synthesized from dietary linolenic acid in the liver (18:3,n-3) (6). DHA supplied by the liver (7) is taken up from the neurovascular unit (blood-brain barrier or blood-inner retina barrier) or from the choriocapillaris (beneath the retina) by the RPE. DHA is then delivered from the RPE to the PRC inner segments through the space called the interphotoreceptor matrix (IPM) (5, 8). The liver drives DHA supply to the nervous system (7). It has been shown that during early postnatal development, administered radiolabeled 18:3 is rapidly taken up by the liver where it is elongated and desaturated to DHA and then supplied by way of the bloodstream to the brain and retina. During synaptogenesis and photoreceptor biogenesis, plasma lipoproteins secreted by the liver are the source of DHA for the nervous system. This systemic bloodstream route (long loop) operates for the morphogenesis and maintenance of excitable membranes (e.g., during constant renewal of photoreceptor disc membranes). The RPE takes DHA from circulating lipoproteins in the choriocapillaris and participates in the DHA recycling from degraded phagosomal phospholipids back to the inner segments of photoreceptors (short loop), following each phagocytic event. The interplay among efficient DHA delivery from the liver, selective uptake by RPE and PRC, and avid retinal retention results in the enrichment of excitable membranes with DHA-phospholipids. In the PRC inner segments, DHA is incorporated at the sn-2, mainly in phosphatidylcholine (PC) that represents more than 50% of PRC phospholipids (9), and is used for the biogenesis of membranes, including synaptic; however, most of it is used for the PRC outer segments (10, 11). DHA phospholipids modulate lipid rafts (12) and physically interact with rhodopsin (13), facilitating conformational changes of rhodopsin, and the associated G proteins are absorbed as photons (14, 15, 16).

In the frog retina, DHA input to the photoreceptors occurs by way of the RPE cells. After passing through the IPM, it is selectively taken into the inner segments of PRC where it is activated and esterified in the sn-2 of a phospholipid. Additionally, rod outer segments occur in two ways: (i) a general diffuse pathway probably common to all FAs, which rapidly labels the entire outer segment and (ii) a specific dense pathway utilized only by DHA-containing phospholipids, which become part of disc membranes along with opsin. The accumulation of newly synthesized basal discs pushes older DHA-laden discs apically until the outer segment tips are shed into the RPE cytoplasm. There, as DHA is released from the disc membranes during phagolysosome degradation, recycling directs DHA back through the IPM, thus conserving this molecule in the retina and permitting it to be selectively taken up by PRC again for the biogenesis of new membranes. The process of DHA handling and trafficking by the retina is specifically orchestrated by a conservation mechanism regulated by RPE cells that ensures adequate DHA for photoreceptors at all times through a short feedback loop from the phagosomes to the IPM.

## Elongation of very long-chain fatty acids-4 and the synthesis of very long-chain PUFAs

ELOVL4 (Elongation of very long-chain fatty acids-4) (17, 18) elongates in the inner segments of PRC 26:6,n-3 derived from DHA or EPA (19, 20). Notwithstanding the low abundance of retinal EPA compared with DHA, EPA is the main substrate for VLC-PUFA formation (20). The resulting VLC-PUFAs became mainly acyl chains in sn-1 of PC. ELOVL4 catalyzes the synthesis of VLC-PUFAs in the retina (10, 21) and testes (22) and generates VLC-saturated FAs in the skin and brain (23, 24). VLC-PUFAs,n-3 (≥ 28 carbons) are at sn-1 of the PC that interacts with rhodopsin (25). The inability of the retina to take up and incorporate DHA leads to homeostatic disruptions and PRC death, as in retinal degenerative diseases (11, 26, 27).

## Adiponectin receptor 1 is necessary for DHA uptake/ELV synthesis in PRC and RPE

RPE cells are necessary for PRC integrity, and damage to these cells participates in retinal degenerations. One of the functions of RPE is to process and recycle DHA during PRC outer segment renewal. Adiponectin receptor 1 (ADIPOR1) is necessary for supplying DHA to the photoreceptors (26). Here, we summarize work that uncovered the structures of ELVs and how the genetic ablation of ADIPOR1 shuts off VLC-PUFA,n-3 and ELV synthesis in the retina.

**Fig. 2:**
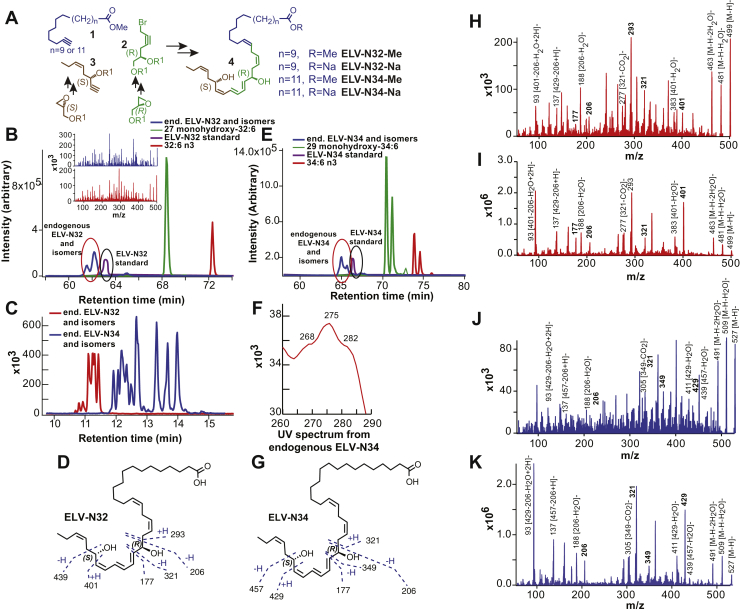
Discovery and structural characterization of ELV-N32 and ELV-N34 in primary human RPE cells in culture. A: ELV-N32 and ELV-N34 were synthesized from three key intermediates (1, 2, and 3), each of which was prepared in stereochemically pure form. The stereochemistry of intermediates 2 and 3 was predefined by using enantiomerically pure epoxide starting materials. The final ELVs (4) were assembled via iterative couplings of intermediates 1, 2, and 3 and were isolated as methyl esters (Me) or sodium salts (Na). B: 32:6n3 (red line), endogenous mono-hydroxy-32:6n3 (green line), and ELV-N32 (blue line) are shown with the ELV-N32 standard (purple). Multiple reaction monitoring of ELV-N32 shows two large peaks eluted earlier than the peak when standard ELV-N32 was eluted, displaying the same fragmentation patterns (shown in the insert spectra), suggesting that they are isomers. C: Chromatogram for full daughter scans for ELV-N32 (red line) and ELV-N34 (blue line). D: Fragmentation pattern of ELV-N32. E: Same features as in (B) for 34:6n3 and ELV-N34. F: UV spectrum of endogenous ELV-N34 showing triene features. G: Fragmentation pattern of ELV-N32. H: Full fragmentation spectra of endogenous ELV-N32 and (I) the ELV-N32 standard shows that all major peaks from the standard match to the endogenous peaks. However, endogenous ELV-N32 has more fragments that do not show up in the standard, suggesting that it includes different isomers. J: For ELV-N34, full fragmentation spectra of endogenous ELV-N34 peaks match up with the standard ELV-N34 (K), also suggesting the existence of ELV-N34 isomers. Reproduced, with permission, from *Scientific Reports* (25). ELV, elovanoid; RPE, retinal pigment epithelium.

**Fig. 3:**
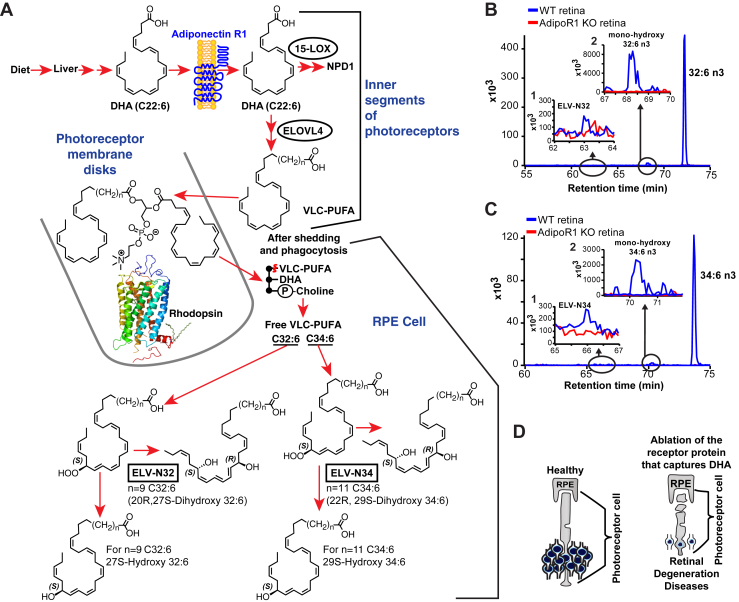
Genetic ablation of adiponectin receptor 1 leads to depletion of VLC-PUFAs and its derivatives in retina. A: Dietary DHA, or DHA derived from dietary 18:3n3, is supplied by the liver and taken by the Adiponectin Receptor 1 (AdipoR1), followed by elongation in the inner segment of photoreceptor cell (PRC) by Elongation of Very Long chain fatty acids-4 (ELOVL4) to VLC-PUFA (very long-chain PUFA) and incorporation into PC molecular species, which contains DHA at sn-2. During daily PRC outer segment renewal, these PC molecular species interact with rhodopsin and, after shedding of the PRC tips and phagocytosis, become part of retinal pigment epithelium (RPE) cells. Uncompensated oxidative stress (UOS) or other disruptors of homeostasis trigger the release of VLC-PUFAs. 32:6n3 and 34:6n3 are depicted generating hydroperoxyl forms, and then elovanoid (ELV)-N32 or ELV-N34, respectively. B: The pool size of free 32:6n3 in retinas of AdipoR1 KO mice (red) is decreased as compared with that in wild type (WT) (blue). Insert (1) shows ELV-N32 in KO (red) and WT (blue); insert (2) shows monohydroxy 32:6n3, the stable derivative of the hydroperoxyl precursor of ELV-N32, in WT (blue) and lack of detectable signal in the KO (red). C: Similarly, the pool size of free 34:6n3 in retinas of AdipoR1 KO mice (red) is decreased as compared with that in WT (blue). Insert (1) shows ELV-N32 in KO (red) and WT (blue); insert (2) shows mono-hydroxy 34:6n3, the stable derivative of the hydroperoxyl precursor of ELV-N34, in WT (blue) and lack of detectable signal in the KO (red). D: RPE cells sustain PRC functional integrity (left); right, the ablation of AdipoR1 switches off DHA availability, and PRC degeneration ensues. Reproduced, with permission, from *Scientific Reports* (25).

The complete structures and stereochemistry of novel ELV-N32 and ELV-N34 were proven using compounds prepared via stereocontrolled total organic synthesis (17). Additional validation of these structural assignments was established by synthesizing deuterium-labeled derivatives (ELV-N32-d2 and ELV-N34-d2) for LC-MS/MS analysis. ELV-N32 and ELV-N34 were prepared by stereocontrolled total chemical synthesis (Fig. 2). Following matching with human primary RPE cell culture media–derived ELVs, the complete structures of ELV-N32 (from a 32C omega-3 PUFA) and ELV-N34 (from a 34C omega-3 PUFA) were confirmed to be ELV-N32 (14*Z*,17*Z*,20*R*,21*E*,23*E*,25*Z*,27*S*,29*Z*)-20,27-dihydroxydo-triaconta-14,17,21,23, 25,29-hexaenoic acid and ELV-N34 (16*Z*,19*Z*,22*R*,23*E*,25*E*,27*Z*,29*S*,31*Z*)-22,29-dihydroxytetra-triaconta-16,19,23,25,27,31-hexaenoic acid (25). Both ELVs and their precursor, the VLC-PUFAs, were clearly formed in RPE cells under uncompensated oxidative stress (UOS). We used m/z 499 → 93 and 499 → 401 MRM transitions for ELV-N32 and m/z 527 → 93 and 527 → 429 transitions for ELV-N34 for detection. For corresponding precursors, we used m/z 483 → 385 for 27-hydroxy-32:6n3 and m/z 511 → 413 for 29–hydroxyl-34:6n3. For further identification, we performed full fragmentation on ELVs and found good matches to the standards (25). ELVs were isolated and characterized. They are stereospecific di-hydroxylated derivatives of VLC-PUFAs (Fig. 3). Moreover, ELV-N32 and ELV-N34 in KO were not synthesized. Evidence that this pathway was downregulated is shown by the fact that mono-hydroxy 32:6n3 and 34:6n3, the stable derivatives of the hydroperoxyl precursors of ELV-N32 and ELV-N34, respectively, were not formed in the KO (25). ELV-N32 and ELV-N34 were found to be secreted from RPE cells when confronted with UOS, implying their involvement in paracrine or autocrine bioactivity. At the same time, the abundance of free 32:6n3 and 34:6n3 in retinas of *AdipoR1* KO mice is considerably decreased (25).

The genetic ablation of ADIPOR1 leads to the depletion of VLC-PUFAs and ELVs in the retina (Fig. 3). UOS or other disruptors of homeostasis trigger the release of 32:6n3 and 34:6n3, hydroperoxyl derivatives, and then ELV-N32 and ELV-N34, respectively (Fig. 3A). The ablation of ADIPOR1 switches off DHA availability, and PRC degeneration ensues (25).

## ELVs target antisurvival and prosurvival proteins with concomitant downregulation of proapoptotic proteins as RPE cells are confronted with UOS

**Fig. 4:**
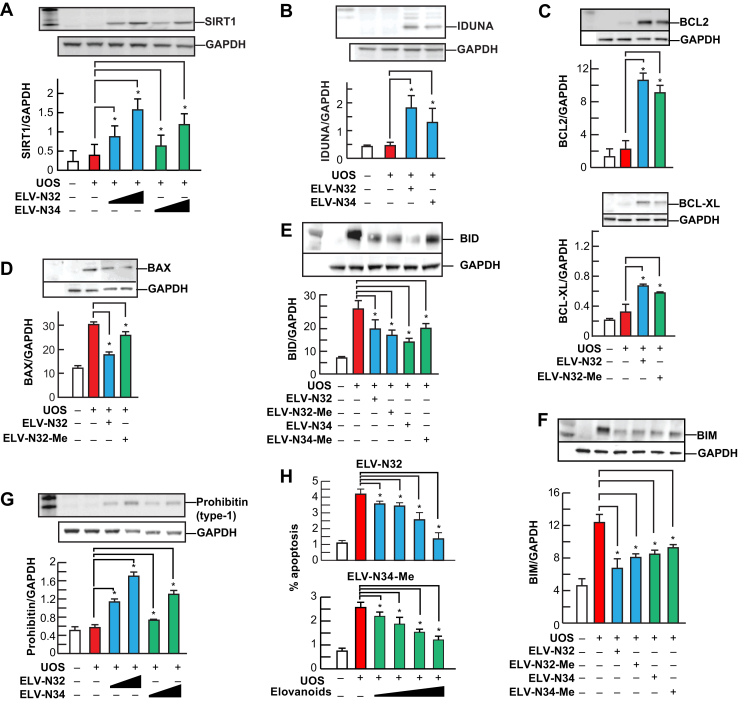
ELV-N32 and ELV-N34 enhance abundance of pro-homeostatic proteins and decrease abundance of cell damaging proteins in RPE cells under UOS. ELV-N32 or ELV-N34 indicates the sodium salt forms, and ELV-N34-Me or ELV-N32-Me indicates the methyl ester forms. ELVs induces the following effects in ARPE-19 cells undergoing UOS: (A) Concentration-dependent (100 and 250 nM) upregulation of SIRT1. The results are the averages of three independent experiments; (B) upregulation of Iduna abundance; (C) increased abundance of antiapoptotic proteins BCL-2 and BCL-XL; (D) decreased abundance of pro-apoptotic proteins BAX, (E) BID and (F) BIM. G: Concentration-dependent (100 and 250 nM) upregulation of Prohibitin (type-1) by ELVs takes place. H: Concentration-dependent (50, 100, 250, and 500 nM) reduction of UOS-induced apoptosis. Error bars, SEM; ∗*P* < 0.05. Reproduced, with permission, from *Scientific Reports* (25). ELV, elovanoid; RPE, retinal pigment epithelium; SIRT1, sirtuin-1; UOS, uncompensated oxidative stress.

ELVs N32 and N34 remarkably enhanced the expression of prosurvival BCL-2 and BCL-XL and downregulated the proapoptotic proteins BAX, BIM, and BID (Fig. 4). Moreover, these mediators also augmented the abundance of prohomeostatic sirtuin-1, iduna, and prohibitin (type-1), a cell-survival protein (28, 29, 30, 31). Sirtuins are involved in retinal disease (32) in aging (33, 34), mitochondrial function (35), and overall homeostasis (36). The other protein targeted is iduna (ring finger protein 146), a PARsylation-directed ring finger E3 ubiquitin ligase that functions in protein quality control, DNA repair, and protection against parthanatos (37, 38), a cell death dependent on poly(ADP-ribose) polymerase-1 (37, 39, 40, 41). poly(ADP-ribose) polymerases mediate the transfer of ADP-ribose from nicotinamide adenine dinucleotide to target proteins and are essential for genomic integrity, the cell cycle, and gene expression (42). The docosanoid neuroprotectin D1 (NPD1) boosts Iduna abundance in RPE cells when exposed to UOS (43).

ELVs upregulated prohibitin (type-1) in these RPE cells undergoing UOS, which is relevant to the biology of senescence and cell survival. Prohibitins are ubiquitous, evolutionarily conserved proteins that form a ring-like, high-molecular-mass complex at the inner membrane of mitochondria and other cellular compartments (29, 30, 31, 44). In addition, they are also involved in energy metabolism, proliferation, apoptosis, and senescence (45). Prohibitin regulates the signaling of membrane transport, control of transcription activation, and the cell cycle, whereas the mitochondrial prohibitin complex stabilizes the mitochondrial genome and modulates mitochondrial dynamics, morphology, biogenesis, and the intrinsic apoptotic pathway (44). Therefore, by eliciting its activity, at least one of the ELV targets, the intracellular abundance of prohibitin (type-1), might provide a mode of exploration to control undesirable consequences of cellular aging.

## ELVs protect RPE cells and sustain PRC integrity

PUFA elongation in the inner segment of PRC by ELOVL4 leads to the biosynthesis of VLC-PUFAs,n-3 and their insertion at the sn-1 of PC for PRC disk membranes. Thereafter, phagocytosis occurs when these unique, plasma-membrane molecular species arrive at the RPE, and if the eye is under conditions of stress, these VLC-PUFAs are cleaved by phospholipase A1 (PLA1) for the synthesis of monohydroxy and dihydroxy VLC-PUFAs (ELVs) to activate ELV synthesis (Fig. 3). Light-induced oxidative stress in mouse retinas triggers the production of free 32:6n3 and 34:6n3, as well as their monohydroxy and dihydroxy derivatives (Fig. 3). Therefore, the lack of the VLC-PUFA,n-3 precursor DHA results in retinal degeneration (26), preceded by a remarkable downregulation of the free VLC-PUFA,n-3 molecular species and ELV biosynthesis. These observations support the concept that VLC-PUFAs,n-3 are precursors of novel bioactive mediators that elicit prohomeostatic protective bioactivity.

Mutant ELOVL4 causes juvenile macular degeneration and other neurological conditions. Among the proposed mechanisms for PRC degeneration caused by mutant ELOVL4 is the loss of its C-terminal endoplasmic reticulum retention signal, leading to protein mislocalization of the truncated ELOVL4 protein that, in turn, causes cellular stress that leads to PRC death. The data presented here suggest an alternative mechanism for the deleterious effects of mutant ELOVL4, which would limit the occurrence of VLC-PUFA,n-3 in the C1 position of phosphatidylcholines (PCs) and sphingolipids. Thus, VLC-PUFAs,n-3 are converted to the corresponding ELVs, which are protective in cell survival under UOS conditions. Under continuous stress, the RPE and retina might need ELVs to sustain the functional integrity of RPE cells and the overall function of PRCs: vision.

The bioactivities of ELV-N32 and ELV-N34 include some unique features. In addition to their potent neuroprotective actions, these lipid mediators (a) are cell-selective, (b) involve a relationship between PRC and RPE cells that is necessary for vision, (c) are derived from VLC-PUFA,n-3, the biosynthesis of which is regulated by a PRC-specific enzyme, ELOVL4, and (d) have precursor FAs (VLC-PUFA,n-3) that are positioned as acyl chains at position C1 of the PC, unlike DHA (the precursor of NPD1), which is incorporated at position C2. Because they are derived from an alternative FA precursor regulated by ELOVL4 and stored at an alternative phospholipid position, the ELVs are likely to involve an alternative activation pathway for exerting their neuroprotective bioactivity in the retina.

Another significant question raised by our novel findings is as follows: Which signaling mechanism targets the novel PC molecular species that appears in RPE cells after shedding and phagocytosis? The PC molecular species in the RPE cell stores precursors of two lipid mediators, DHA in the C2 position and VLC-PUFAs 32:6n3 or 34:6n3 (the precursors of ELV-N32 and ELV-N34) in the C1 position. The PC molecular species is targeted for the release of the acyl chains at C1 and C2 when confronted with UOS, as in the onset of retinal degenerations. The new ELVs reported here provide a novel autocrine/paracrine prohomeostatic RPE signaling that aims to sustain PRC and RPE cell integrity, thus revealing the potential for developing novel therapeutic approaches for retinal degenerations.

## Membrane-type frizzled-related protein and ADIPOR1 preserve PRC integrity by the regulated uptake and distribution of DHA

The genetic deletion of the *Adipor1* gene blocks the ability to take up and incorporate DHA in the PRC and RPE, resulting in the loss of PRC and retinal degeneration (26, 46). Most recently, it was found that a second protein, membrane-type frizzled-related protein (MFRP), is also engaged in DHA retention. MFRP is a glycosylated transmembrane protein with an extracellular frizzled-related cysteine-rich domain (47, 48) that recognizes frizzled Wnt receptors (48, 49), plays a role in cell fate and development (47, 49), and is expressed in the RPE and ciliary bodies (48). Moreover, gene mutations encoding this protein are associated with PRC degeneration. *Mfrp*^*rd6*^ results from a 4 bp deletion in a splice donor sequence leading to exon 4 skipped and finally a truncated protein and retinal degeneration (48). These mice present subretinal space macrophage infiltration (50, 51, 52) similar to flecked retinal diseases (53) as human *retinitis punctata albescens* (52, 54). MFRP protein function still is not clearly defined (46, 55). It is of interest that mutations of *Adipor1* display phenotypic analogies with those of *Mfrp* (46). The *Mfrp* ^*rd6*^ mice lack RPE ADIPOR1 (46). A question here is whether the RPE ADIPOR1 shortage might trigger the onset of photoreceptor degeneration in *rd6* mice (46).

**Fig. 5:**
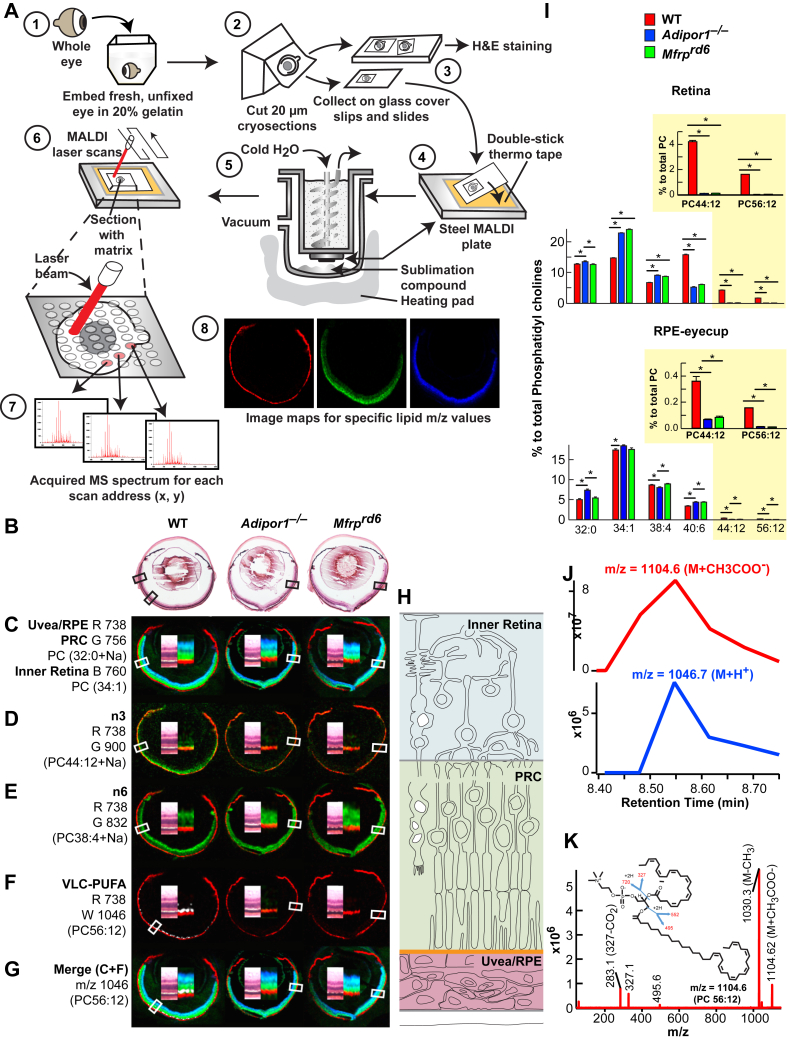
MALDI IMS reveals loss of PC containing 22:6 and VLC-PUFAs in the outer nuclear layer of *Mfrp*^*rd6*^ and *Adipor1*^*−/−*^ retinas. A: Whole eyes embedded in gelatin (1), frozen, cryo-sectioned, 20 μm, collected on alternating glass slides (for H&E) and coverslips (for MALDI). Coverslips attached to MALDI plates placed within sublimation chamber, matrix (56) (2,5-dihydroxybenzoic acid, DHB) applied for positive ion mode analysis MALDI Synapt G2-Si MS. Sections rastered by laser, 355 nm, 2000 Hz. Scanning control (30 μm, horizontal and vertical movement) and analysis. Image spot consisted of a collection of one second of data acquisition. Image processing (8). B: H&E sections of adjacent MALDI section for WT, *Adipor1*^*−/−*^, and the *Mfrp*^*rd6*^ mice. C: Lipid markers for uvea/RPE (m/z 738, red, R), PRC (m/z 756, green, G), and inner retina (m/z 760, blue, B). D: n-3 containing PC (22:6/22:6), m/z 900 (green) is present in WT but absent in *Adipor1*^*−/−*^ and *Mfrp*^*rd6*^ retinas. E: n-6-containing phospholipids compensate D. Here, PC (18:0/20:4 n-6, m/z 832, green) is reduced in WT PRC, but still abundant within the inner retina while present within PRC of *Adipor1*^*−/−*^ and *Mfrp*^*rd6*^. F: 22:6 is elongated to produce VLC-PUFAs. WT retina contains 34:6 within PC(34:6/22:6); 34:6 is indicated by the white label, whereas the *Adipor1*^*−/−*^ and the *Mfrp*^*rd6*^ mice show no 34:6. G: The merge of C and F illustrates the location of 34:6 just inside the uvea/RPE marker (red) at the outer region of PRC (green) in WT, but no label within mutant retinas (absence of 22:6). H: Diagram on retinal location of c color markers. Double inset within each retina image is an enlargement of MALDI label and the corresponding area from H&E in b, indicated by boxes. N = 3 for each condition. I: LC-MS/MS quantitative PC molecular species distributions in bar graphs for retina (top) and RPE (bottom). 22:6-containing PCs, which are abundant in PRC outer segments, are reduced in *Adipor1*^*−/−*^ and *Mfrp*^*rd6*^ retinas and RPE, especially PC 44:12 and PC 56:12 (yellow insets). Student's *t*-test (∗*P* < 0.05). J: Retention time of negative ion mode of PC 56:12 (M + CH3COO) at m/z = 1104.6 (top) matches with positive ion mode (M + H+) at m/z = 1047.7, confirming that m/z = 1046.7 measured in MALDI is from PC 56:12 (M + H+). K: Full fragmentation spectrum of PC 56:12 (M + CH3COO−) measured in negative mode shows composition of PC 56:12 (34:6/22:6). Reproduced, with permission, from the *Federation of American Societies for Experimental Biology* (57). IMS, imaging mass spectrometry; PRC, photoreceptor cell; RPE, retinal pigment epithelium; VLC-PUFA, very long-chain PUFA.

**Fig. 6:**
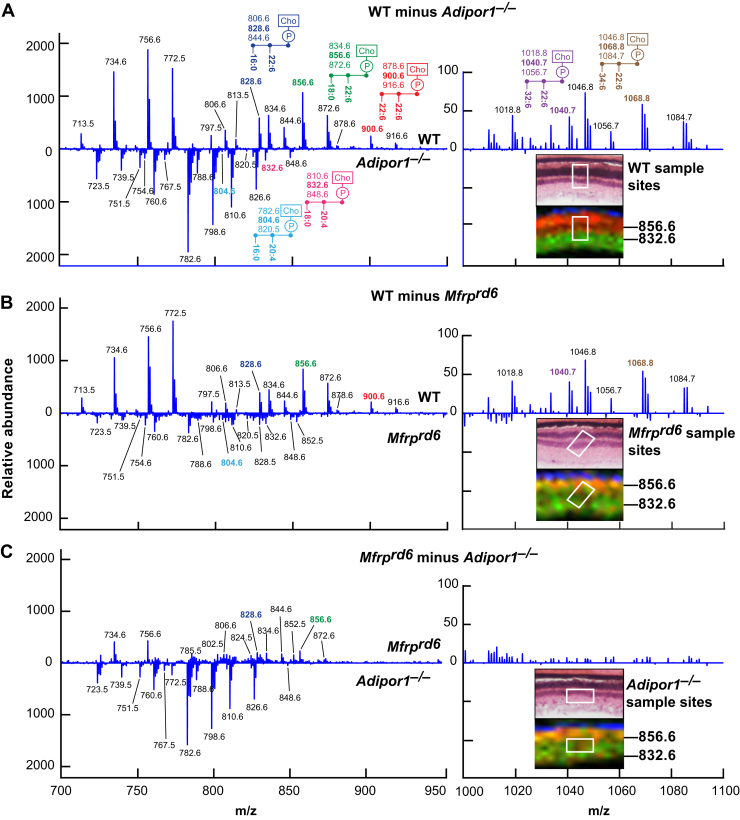
Differential MALDI spectra reveal compensatory PCs generated in *Adipor1*^*−/−*^ and the *Mfrp*^*rd6*^ retina. Difference spectra show relative abundances detected by MALDI IMS. A: Molecules more abundant in WT retina versus *Adipor1*^*−/−*^ retina are presented in the upper part of the graph, while molecules more abundant in the *Adipor1*^*−/−*^ retina are displayed at the bottom. 22:6- and/or VLC-PUFA–containing PCs are much more abundant in WT while 20:4-containing PCs are increased in the mutant retina. The regions from which the spectra are extracted are shown in the insets H and E (top) and MALDI (bottom), showing PC40:6 (m/z = 856.6, red) and PC38:4 (m/z = 832.6, green). B: The difference spectra of *Mfrp*^*rd6*^ to WT. C: The difference spectra of *Adipor1*^*−/−*^ to *Mfrp*^*rd6*^. Inset images show that in both mutants, the PRC layers contain both PCs 40:6 and 38:4 (yellow), whereas WT has clear separation of the two PCs species between PRC layer and inner retina layer. Reproduced, with permission, from the *Federation of American Societies for Experimental Biology* (57). IMS, imaging mass spectrometry; PRC, photoreceptor cell; VLC-PUFA, very long-chain PUFA.

**Fig. 7:**
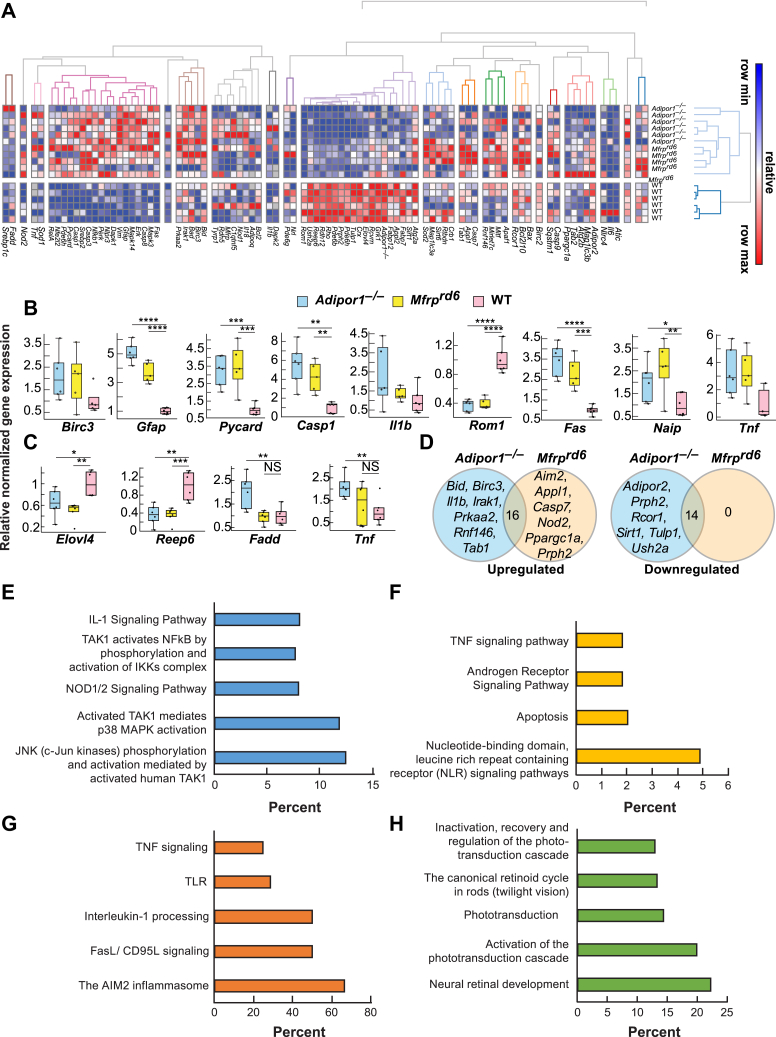
The expression of inflammatory markers is increased and visual system markers decreased in *Adipor1*^*−/−*^ and *Mfrp*^*rd6*^. A: Heat map illustrating the hierarchical clustering of 88 target genes for the retinas of *Adipor1*^*−/−*^ (n = 6), *Mfrp*^*rd6*^ (n = 5), and WT mice (n = 6). Color key represents row scaling of normalized fold-change (FC) expression values from RT-qPCR. B: Box-plots representation of selected genes for the retinas with their FC below −2 or above +2, relative to the WT. Boxes denote median values with upper and lower quartiles, and whiskers, minimum and maximum outliers. C: Box-plots representation of selected genes for the RPE-eyecup samples with their FC below −2 or above +2, relative to WT. D: Venn diagrams of differentially expressed genes within *Adipor1*^*−/−*^ and *Mfrp*^*rd6*^ data sets that are upregulated or downregulated in a statistically significant manner in the retina compared with the WT. The number in the intersection represents the differentially expressed genes that are common between the two data sets. Top pathways resulting from the upregulation of genes in *Adipor1*^*−/−*^ retina (E) or *Mfrp*^*rd6*^ retina (F) compared with the WT or common upregulated (G) or downregulated (H) genes in both mutant retinas. Student's *t*-test was used. ∗*P* ≤ 0.05, ∗∗*P* ≤ 0.01, ∗∗∗*P* ≤ 0.001, ∗∗∗∗*P* ≤ 0.0001, NS = not significant. Error bars represent standard deviation. When there was no statistical difference in the mutant data sets compared to the WT, statistic bars were not added. Reproduced, with permission, from the *Federation of American Societies for Experimental Biology* (57). RPE, retinal pigment epithelium.

The mutations of *Mfrp* and *Adipor1* yield a similar transcriptomic signature, lipidome conformation, and cell function (Fig. 5, Fig. 6, Fig. 7). Sensitive and specific MALDI imaging mass spectrometry has allowed mapping of the untargeted in situ spatial distribution of the molecular species of phospholipids, complemented with LC-MS/MS quantification and detailed characterization. Optical coherence tomography imaging and electroretinograms (ERGs) have allowed assessment of retina structure and function. Transcriptomic approaches define gene signatures involved in PRC degeneration in the *Mfrp*^*rd6*^ mouse and establish analogies and differences with the *Adipor1*^*−/−*^ mouse, preceding PRC degeneration (Figs. 6 and 7).

It is of interest that a single amino acid mutation of *Adipor1* has been found to underlie a sizable group of retinitis pigmentosa patients (58, 59). Also, polymorphisms of *Adipor1* occur in age-related macular degeneration (AMD) (60) and other forms of retinal degenerations.

The deletion of *Mfrp* or *Adipor1* results in (a) flecked retina and the onset of PRC death; (b) ERG attenuation; (c) perturbed ability to take up and incorporate DHA in RPE and PRC, which consequently leads to a perturbed retinal lipidome; and (d) transcriptomic signature modifications with enhanced proinflammatory cytokine pathways. However, each mutation has some differences (57). Overall, MFRP and ADIPOR1 preserve PRC integrity by a regulated enrichment and distribution of DHA. The modification of DHA pool size and distribution affects the synthesis of VLC-PUFAs that supply the precursors for prohomeostatic ELVs. Therefore, the maintenance of a balanced DHA/VLC-PUFA relies on MFRP and ADIPOR1, which safeguard proper attainment and distribution of key lipids from the RPE to the PRC that are necessary to sustain sight and function at the onset of AMD and other retinal degenerations.

To maintain PRC functions, membrane phospholipids provide an environment for phototransduction to proceed and precursors of biologically active lipid mediators in the inner segment of PRC, as well as in the RPE (25, 27). Therefore, a tight balance on retinal lipidome homeostasis must be maintained because UOS affects highly unsaturated FAs, forming DHA protein adducts that contribute to retinal degeneration (61). Overall, a healthy retinal lipidome is essential for PRC function within a highly stressful environment of bright light, high-oxygen demands, and an abundance of PUFAs, which are highly susceptible to oxidative damage (8, 27).

## ELVs protect RPE cells and, in turn, support PRC integrity

DHA or EPA elongation in the inner segment of PRC by ELOVL4 leads to VLC-PUFAs,n-3 biosynthesis and esterification at the sn-1 of PC. However, under conditions of stress, these VLC-PUFAs are cleaved by PLA1 for the synthesis of dihydroxy VLC-PUFAs (ELVs) (Fig. 3A). Light-induced oxidative stress in mouse retinas triggers the production of free 32:6n3 and 34:6n3, as well as their monohydroxy and dihydroxy derivatives (Fig. 3A). In AdipoR1 KO mice, no detectable amounts of these molecules were found (Fig. 3B, C, red curves). Also, the lack of the VLC-PUFA,n-3 precursor DHA results in retinal degeneration (Fig. 3D) (26), preceded by a remarkable downregulation of the free VLC-PUFA,n-3 molecular species and ELV biosynthesis. These observations support the hypothesis that VLC-PUFAs,n-3 are precursors of novel bioactive mediators that elicit prohomeostatic protective bioactivity.

## ELVS Target PRC/RPE senescence gene programming, the senescence secretory phenotype, and the extracellular matrix: implications to AMD, Alzheimer's disease, and other pathologies

**Fig. 8:**
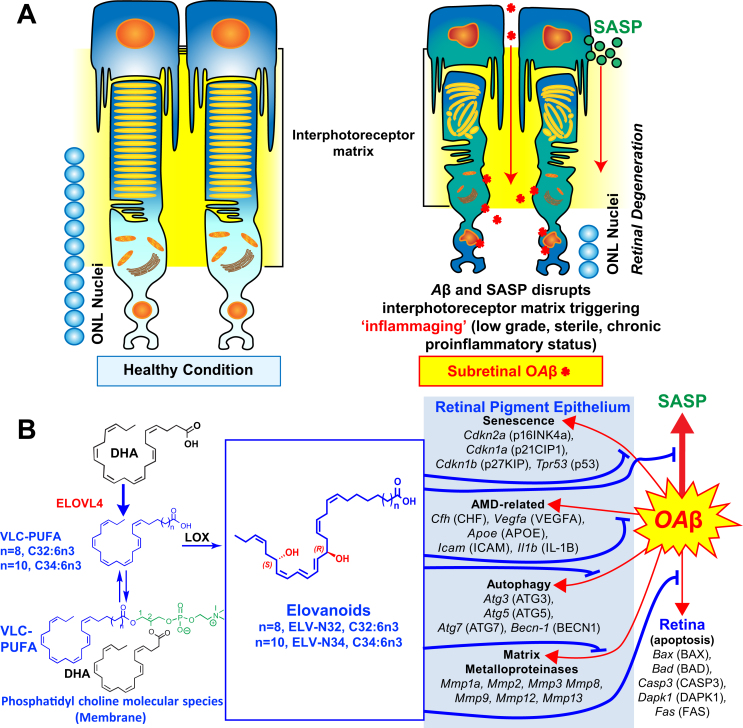
ELVs effect on oligomeric amyloid-β (OAβ)-induced RPE and PRC damage. A: OAβ induces a senescence gene program and disrupts RPE tight junctions. OAβ penetrates the retina, causing PRC cell death in our in vivo WT mice study, as reflected in less outer nuclear layer (ONL) nuclei (Fig. 5 from Do *et al.* (62)). OAβ activates the senescence-associated secretome (SASP) that contributes to perturbing the interphotoreceptor matrix (IPM), triggering inflammaging in PRC and also likely in Mueller glia, which limits the IPM. Therefore, senescence paracrine expression takes place. ELVs restore RPE morphology and PRC integrity. B: OAβ induces expression of senescence, autophagy, matrix metalloproteinases, and age-related macular degeneration (AMD)-related genes in the RPE and apoptosis genes in retina in addition to p16^INK4a^ protein abundance. ELVs downregulated the OAβ-gene inductions and p16^INK4a^ protein abundance. Pathways for the ELV synthesis are outlined. Reproduced, with permission, from the *Proceedings of the National Academy of Sciences of the United States* (62). ELV, elovanoid; PRC, photoreceptor cell; RPE, retinal pigment epithelium.

Oligomeric amyloid-β (OAβ) triggers apoptosis-mediated cell death in PRC and in the brain. We review here how ELVs prevent both OAβ-induced senescence gene programming in RPE as well as OAβ-induced PRC apoptosis (62). Moreover, sluggish prohomeostatic lipid mediator pathways preceding PRC death in the 5xFAD mice take place. At the same time, decreased content of PC molecular species in RPE (particularly those containing VLC-PUFAs) and in retina (those containing DHA and VLC-PUFAs) takes place. The pool size of free VLC-PUFAs and stable derivatives of precursors 27- and 29-monohydoxy and of ELV-N32 and ELV-N34, respectively, were depleted in 5xFAD mice (62). These changes reflect retina insufficiencies in key enzymes of the synthesis of prohomeostatic/neuroprotective NPD1 and ELVs without the evidence of PRC damage or loss but clearly display ERG impairments. ELV administration effectively counteracts cytotoxicity against subretinal administered OAβ in WT mice, leading to RPE tight junction disruptions followed by PRC cell death. OAβ markedly activates a senescence gene program reflected by the enhanced expression of *Cdkn2a*, *Mmp1a*, *Trp53*, *Cdkn1a*, *Cdkn1b*, *Il-6*, and of senescence-associated secretory phenotype (SASP) secretome, followed by RPE and PRC death (62). ELV-N32 and ELV-N34 blunt these events and elicit protection to both cells. The RPE cell is terminally differentiated and originated from the neuroepithelium. In this connection, senescent neurons in aged mice and Alzheimer's disease (AD) models (50) and astrocytes (51, 52) also express senescence programs and develop secretory SASP that fuel neuroinflammation in nearby cells (53, 54, 55). This is likely the case in the PRC/RPE, where neighbor cells may be targeted by SASP neurotoxic actions, inducing PRC paracrine senescence. Therefore, SASP from RPE cells may be autocrine and paracrine, altering the homeostasis of the extracellular microenvironment, the IPM in this case (Fig. 8). Furthermore, ELVs restore the expression of the extracellular matrix remodeling matrix metalloproteinases altered by OAβ. As an outcome, an inflammaging milieu (low-grade, chronic inflammatory process) contributes to the loss of function associated with aging (58), age-related pathologies (58), AMD, and likely AD.

## Concluding comments and forward-looking perspective on questions that remain to be answered

Physiologically, DHA is beneficial and required for RPE and PRC function. A big question that remains is how all of this fits together. Here, we review data that support that DHA is important for retina function (5, 63, 64, 65). Up to 50% of the PRC's phospholipids are a molecular species of PC, which contain DHA (22C) at the sn-2 and VLC-PUFAs,n-3 (≥ 28 carbons, derived from DHA) at the sn-1 position. These PCs are strongly associated with opsin (10, 66, 67, 68, 69), and the high degree of unsaturation of these PC molecular species creates a fluid environment in the PRC disk membrane for conformational changes required by opsin function in phototransduction. After the daily shedding of PRC tips and RPE phagocytosis, disk membrane packets are disassembled in the phagolysosomal system, and the DHA-containing molecules are recycled back to the PRC inner segments for the biosynthesis of PC and reinsertion into new disk membranes. Importantly, a DHA-restricted diet in subhuman primates (70, 71) takes several generations to deplete retinal DHA. DHA depletion studies in rodents have shown that reduced DHA results in decreased efficiency in rhodopsin's ability to capture a photon and initiate the visual cycle, and the efficiency of the associated G-protein is also reduced.

DHA is necessary for the synthesis of the prohomeostatic/neuroprotective mediators: NPD1 and ELVs (Fig. 1). When VLC-PUFAs are released by PLA1, they can enter into the pathway to form ELVs. PLA2 cleavage of DHA leads to the synthesis of docosanoids; the most prominently studied is NPD1. The other side of the coin is that, under pathological conditions, DHA peroxidation and DHA-derived protein adducts are participants of retinal degeneration. The process of DHA peroxidation leads to the conversion of protein adducts and results in PRC damage (72). Thus, enhanced ferroptosis and peroxidation products accumulate, and the RPE cells are affected initially with subsequent PRC demise because they depend on the RPE for survival. Two keys in this dependency are the successful elongation of DHA to VLC-PUFAs and the readiness to form protective ELVs from the VLC-PUFAs.

Dietary DHA (or derived from dietary 18:3n3) is supplied to tissues by the liver and captured by AdipoR1, followed by elongation in the inner segment of PRCs by ELOVL4 to VLC-PUFA,n-3 and incorporation into PC molecular species, which are also endowed with DHA. ELOVL4 uses EPA as a preferred substrate (20), in spite of the fact that the abundance of EPA is very low in the retina compared with DHA. Retroconversion of DHA to EPA in peroxisomes and EPA generated by this reaction might form the 26C PUFA that is the substrate for ELOVL4 (20). During daily PRC outer segment renewal, these PC molecular species interact with rhodopsin (66) and, after shedding and phagocytosis, become part of the RPE cells. UOS or other disruptors of homeostasis triggers the release of VLC-PUFAs. 32:6n3 and 34:6n3 generate a hydroperoxyl intermediate and then an ELV-N32 or ELV-N34, respectively.

ELVs are the first known lipid mediators from VLC-PUFA,n-3 that are biosynthetic derivatives of elongase ELOVL4. The structure and stereochemistry of ELVs N32 and N34 were established (Fig. 2). ELV synthesis is eliminated in mice retinas with genetically ablated AdipoR1, implying the requirement of DHA for their formation (25). ELVs are biosynthesized in human RPE cells and have protective functions in RPE cells undergoing UOS (25). The protein targets of ELVs in RPE cells uncovered prohomeostatic functions and previously unknown pathways for preserving PRC integrity. ELV biosynthesis comprises an alternative activation pathway from VLC-PUFA,n-3 located at sn-1 of PC, whereas DHA is located at sn-2. It is of interest that the storage of the ELV precursors seems to be mainly PC, because sphingomyelin or other phospholipids, such as PE and PS, do not seem to be involved (25).

Overall, VLC-PUFA,n-3 availability is needed to sustain prohomeostatic events and RPE cytoprotection. The synthesis of the novel lipids biosynthesized under these conditions was defined, including the S-precursor and the stable analogs of hydroxyl-derivatives (8). The novel ELV bioactive lipids (ELV-N32 and ELV-N34) involve the prior release of either 32:6n3 or 34:6n3 from the C1 position of PC. Because this phospholipid molecular species also has DHA at sn-2, we suggest that NPD1 also can be made from the same phospholipid reservoir. Thus, we review here a different signal bifurcation mechanism to sustain PRC and RPE cell integrity, supported by the observation that genetic ablation of AdipoR1 results in the depletion of these molecular species.

SASP from RPE cells may be autocrine and paracrine, altering the homeostasis of the IPM microenvironment (Fig. 8) as a consequence, creating an inflammatory milieu that contributes to loss of function associated with aging (58), age-related pathologies (58), AMD, and likely AD. Furthermore, ELVs restore expression of extracellular matrix remodeling matrix metalloproteinases altered by OAβ treatment, pointing to an additional disturbance in the IPM. The inflammation set in motion may be a low-grade, sterile, chronic proinflammatory condition similar to inflammaging that is also linked to senescence of the immune system (58).

Among the questions that remain unanswered are the following: (a) Which signals trigger VLC-PUFA release by PLA1? Is there a concerted regulation to activate the ELVs pathway with signals that modulate PLA2 cleavage leading to the synthesis of docosanoids (e.g., NPD1)? Do neurotrophins modulate these pathways, as in the case of pigment epithelium-derived factor (and other neurotrophins) for NPD1 synthesis (2)? Are there synergies in the biological activity of NPD1 (or other docosanoids) and ELVs? These divergent pathways would involve regulation by PRC-specific phospholipases A1 and A2 as a novel neuroprotective event. (b) Are the DHA requirements during function or repair (after ischemic injury or neurodegenerative disorders) met by a signal sent by the brain or retina to the liver to evoke the secretion of DHA-containing lipoproteins (7)? (c) It is also unclear whether the ELVs that targeted gene transcription (Fig. 8) inform novel unifying regulatory mechanisms to sustain health span during aging and neurodegenerative diseases. (d) Is there a concerted expression/function of ADIPOR1 and MFRP in so far as retention and metabolism of ELVs and docosanoids? (e) Do VLC-PUFAs recycle like DHA between PRC and RPE? (f) Do VLC-PUFAs,n-3 undergo peroxidation and formation of VLC-PUFA-derived protein adducts that fuel retinal degeneration, as is the case with DHA?

This overview summarizes lessons learned from evolving novel molecular targets for neuroprotection. The N32 and N34 ELVs provide a novel autocrine/paracrine prohomeostatic signaling for neuroprotection. As an example, SASP from RPE cells may be autocrine and paracrine, altering the homeostasis of the IPM microenvironment (Fig. 8) as a consequence, creating an inflammatory milieu that contributes to loss of function associated with aging (58), age-related pathologies (58), AMD, and likely AD. Several forms of retinal degenerative diseases, including retinitis pigmentosa and other inherited retinal degenerations, may underlie these mechanisms, and ELVs might halt the onset or slow down disease progression. Although further research is needed, overall, there is potential for ELVs as therapeutic avenues and insights for the development of new therapeutics for AMD, AD, and other pathologies.

## Conflict of interest

The author is the founder of two startup companies that have exclusively licensed technologies from LSUHSC involving elovanoids and related lipids for clinical applications: NeuResto Therapeutics, LLC (http://www.neuresto.com/) and CurVirBiotech, LLC (web site in construction).

## References

[1] Serhan C.N. (2017). Treating inflammation and infection in the 21st century: new hints from decoding resolution mediators and mechanisms. FASEB J..

[2] Bazan N.G. (2007). Homeostatic regulation of photoreceptor cell integrity: significance of the potent mediator neuroprotectin D1 biosynthesized from docosahexaenoic acid: the Proctor Lecture. Invest. Ophthalmol. Vis. Sci..

[3] Bazan N.G. (2009). Cellular and molecular events mediated by docosahexaenoic acid-derived neuroprotectin D1 signaling in photoreceptor cell survival and brain protection. Prostaglandins Leukot. Essent. Fatty Acids.

[4] Calder P.C. (2017). Omega-3 fatty acids and inflammatory processes: from molecules to man. Biochem. Soc. Trans..

[5] Rodriguez de Turco E.B., Gordon W.C., Bazan N.G. (1991). Rapid and selective uptake, metabolism, and cellular distribution of docosahexaenoic acid among rod and cone photoreceptor cells in the frog retina. J. Neurosci..

[6] Metherel A.H., Irfan M., Chouinard-Watkins R., Trépanier M.-O., Stark K.D., Bazinet R.P. (2019). DHA cycling halves the DHA supplementation needed to maintain blood and tissue concentrations via higher synthesis from ALA in Long-Evans rats. J. Nutr..

[7] Scott B.L., Bazan N.G. (1989). Membrane docosahexaenoate is supplied to the developing brain and retina by the liver. PNAS.

[8] Bazan N.G., Molina M.F., Gordon W.C. (2011). Docosahexaenoic acid signalolipidomics in nutrition: significance in aging, neuroinflammation, macular degeneration, Alzheimer's, and other neurodegenerative diseases. Annu. Rev. Nutr..

[9] Fliesler S.J., Anderson R.E. (1983). Chemistry and metabolism of lipids in the vertebrate retina. Prog. Lipid Res..

[10] Aveldaño M.I. (1987). A novel group of very long chain polyenoic fatty acids in dipolyunsaturated phosphatidylcholines from vertebrate retina. J. Biol. Chem..

[11] Bazan N.G. (2006). Cell survival matters: docosahexaenoic acid signaling, neuroprotection and photoreceptors. Trends Neurosci..

[12] Wassall S.R., Leng X., Canner S.W., Pennington E.R., Kinnun J.J., Cavazos A.T., Dadoo S., Johnson D., Heberle F.A., Katsaras J., Shaikh S.R. (2018). Docosahexaenoic acid regulates the formation of lipid rafts: A unified view from experiment and simulation. Biochim. Biophys. Acta Biomembr.

[13] Soubias O., Teague W.E., Gawrisch K. (2006). Evidence for specificity in lipid-rhodopsin interactions. J. Biol. Chem..

[14] Sánchez-Martín M.J., Ramon E., Torrent-Burgués J., Garriga P. (2013). Improved conformational stability of the visual G protein-coupled receptor rhodopsin by specific interaction with docosahexaenoic acid phospholipid. Chembiochem.

[15] Litman B.J., Niu S.L., Polozova A., Mitchell D.C. (2001). The role of docosahexaenoic acid containing phospholipids in modulating G protein-coupled signaling pathways: visual transduction. J. Mol. Neurosci..

[16] Mitchell D.C., Niu S.-L., Litman B.J. (2003). Enhancement of G protein-coupled signaling by DHA phospholipids. Lipids.

[17] Lagali P.S., Liu J., Ambasudhan R., Kakuk L.E., Bernstein S.L., Seigel G.M., Wong P.W., Ayyagari R. (2003). Evolutionarily conserved ELOVL4 gene expression in the vertebrate retina. Invest. Ophthalmol. Vis. Sci..

[18] Hopiavuori B.R., Anderson R.E., Agbaga M.-P. (2019). ELOVL4: Very long-chain fatty acids serve an eclectic role in mammalian health and function. Prog. Retin. Eye Res..

[19] Suh M., Clandinin M.T. (2005). 20:5n-3 but not 22:6n-3 is a preferred substrate for synthesis of n-3 very-long- chain fatty acids (C24-C36) in retina. Curr. Eye Res..

[20] Yu M., Benham A., Logan S., Brush R.S., Mandal M.N.A., Anderson R.E., Agbaga M.-P. (2012). ELOVL4 protein preferentially elongates 20:5n3 to very long chain PUFAs over 20:4n6 and 22:6n3. J. Lipid Res..

[21] Agbaga M.-P., Mandal M.N.A., Anderson R.E. (2010). Retinal very long-chain PUFAs: new insights from studies on ELOVL4 protein. J. Lipid Res..

[22] Oresti G.M., Ayuza Aresti P.L., Gigola G., Reyes L.E., Aveldaño M.I. (2010). Sequential depletion of rat testicular lipids with long-chain and very long-chain polyenoic fatty acids after X-ray-induced interruption of spermatogenesis. J. Lipid Res..

[23] Cameron D.J., Tong Z., Yang Z., Kaminoh J., Kamiyah S., Chen H., Zeng J., Chen Y., Luo L., Zhang K. (2007). Essential role of Elovl4 in very long chain fatty acid synthesis, skin permeability barrier function, and neonatal survival. Int. J. Biol. Sci..

[24] Monroig Ó., Rotllant J., Cerdá-Reverter J.M., Dick J.R., Figueras A., Tocher D.R. (2010). Expression and role of Elovl4 elongases in biosynthesis of very long-chain fatty acids during zebrafish Danio rerio early embryonic development. Biochim. Biophys. Acta Mol. Cell Biol. Lipids.

[25] Jun B., Mukherjee P.K., Asatryan A., Kautzmann M.-A., Heap J., Gordon W.C., Bhattacharjee S., Yang R., Petasis N.A., Bazan N.G. (2017). Elovanoids are novel cell-specific lipid mediators necessary for neuroprotective signaling for photoreceptor cell integrity. Sci. Rep..

[26] Rice D.S., Calandria J.M., Gordon W.C., Jun B., Zhou Y., Gelfman C.M., Li S., Jin M., Knott E.J., Chang B., Abuin A., Issa T., Potter D., Platt K.A., Bazan N.G. (2015). Adiponectin receptor 1 conserves docosahexaenoic acid and promotes photoreceptor cell survival. Nat. Commun..

[27] Bazan N.G. (2018). Docosanoids and elovanoids from omega-3 fatty acids are pro-homeostatic modulators of inflammatory responses, cell damage and neuroprotection. Mol. Aspects Med..

[28] Mukherjee P.K., Chawla A., Loayza M.S., Bazan N.G. (2007). Docosanoids are multifunctional regulators of neural cell integrity and fate: significance in aging and disease. Prostaglandins Leukot. Essent. Fatty Acids.

[29] Li L., Guo J.-D., Wang H.-D., Shi Y.-M., Yuan Y.-L., Hou S.-X. (2015). Prohibitin 1 gene delivery promotes functional recovery in rats with spinal cord injury. Neuroscience.

[30] Sripathi S.R., Sylvester O., He W., Moser T., Um J.-Y., Lamoke F., Ramakrishna W., Bernstein P.S., Bartoli M., Jahng W.J. (2016). Prohibitin as the molecular binding switch in the retinal pigment epithelium. Protein J..

[31] Sripathi S.R., He W., Sylvester O., Neksumi M., Um J.-Y., Dluya T., Bernstein P.S., Jahng W.J. (2016). Altered cytoskeleton as a mitochondrial decay signature in the retinal pigment epithelium. Protein J..

[32] Balaiya S., Abu-Amero K.K., Kondkar A.A., Chalam K.V. (2017). Sirtuins expression and their role in retinal diseases. Oxid. Med. Cell Longev..

[33] van de Ven R.A.H., Santos D., Haigis M.C. (2017). Mitochondrial sirtuins and molecular mechanisms of aging. Trends Mol. Med..

[34] Grabowska W., Sikora E., Bielak-Zmijewska A. (2017). Sirtuins, a promising target in slowing down the ageing process. Biogerontology.

[35] Hershberger K.A., Martin A.S., Hirschey M.D. (2017). Role of NAD+ and mitochondrial sirtuins in cardiac and renal diseases. Nat. Rev. Nephrol..

[36] Jokinen R., Pirnes-Karhu S., Pietiläinen K.H., Pirinen E. (2017). Adipose tissue NAD+-homeostasis, sirtuins and poly(ADP-ribose) polymerases -important players in mitochondrial metabolism and metabolic health. Redox Biol..

[37] Kang H.C., Lee Y.-I., Shin J.-H., Andrabi S.A., Chi Z., Gagné J.-P., Lee Y., Ko H.S., Lee B.D., Poirier G.G., Dawson V.L., Dawson T.M. (2011). Iduna is a poly(ADP-ribose) (PAR)-dependent E3 ubiquitin ligase that regulates DNA damage. Proc. Natl. Acad. Sci. U.S.A..

[38] Andrabi S.A., Kang H.C., Haince J.-F., Lee Y.-I., Zhang J., Chi Z., West A.B., Koehler R.C., Poirier G.G., Dawson T.M., Dawson V.L. (2011). Iduna protects the brain from glutamate excitotoxicity and stroke by interfering with poly(ADP-ribose) polymer-induced cell death. Nat. Med..

[39] Zhang J., Li X., Kwansa H., Kim Y.T., Yi L., Hong G., Andrabi S.A., Dawson V.L., Dawson T.M., Koehler R.C., Yang Z.-J. (2017). Augmentation of poly(ADP-ribose) polymerase-dependent neuronal cell death by acidosis. J. Cereb. Blood Flow Metab..

[40] Lee Y., Karuppagounder S.S., Shin J.-H., Lee Y.-I., Ko H.S., Swing D., Jiang H., Kang S.-U., Lee B.D., Kang H.C., Kim D., Tessarollo L., Dawson V.L., Dawson T.M. (2013). Parthanatos mediates AIMP2-activated age-dependent dopaminergic neuronal loss. Nat. Neurosci..

[41] Andrabi S.A., Kim N.S., Yu S.-W., Wang H., Koh D.W., Sasaki M., Klaus J.A., Otsuka T., Zhang Z., Koehler R.C., Hurn P.D., Poirier G.G., Dawson V.L., Dawson T.M. (2006). Poly(ADP-ribose) (PAR) polymer is a death signal. Proc. Natl. Acad. Sci. U.S.A..

[42] Krietsch J., Rouleau M., Pic É., Ethier C., Dawson T.M., Dawson V.L., Masson J.-Y., Poirier G.G., Gagné J.-P. (2013). Reprogramming cellular events by poly(ADP-ribose)-binding proteins. Mol. Aspects Med..

[43] Belayev L., Mukherjee P.K., Balaszczuk V., Calandria J.M., Obenaus A., Khoutorova L., Hong S.-H., Bazan N.G. (2017). Neuroprotectin D1 upregulates Iduna expression and provides protection in cellular uncompensated oxidative stress and in experimental ischemic stroke. Cell Death Differ.

[44] Nijtmans L.G., de Jong L., Artal Sanz M., Coates P.J., Berden J.A., Back J.W., Muijsers A.O., van der Spek H., Grivell L.A. (2000). Prohibitins act as a membrane-bound chaperone for the stabilization of mitochondrial proteins. EMBO J..

[45] Bourassa C.V., Raskin S., Serafini S., Teive H.A.G., Dion P.A., Rouleau G.A. (2015). A new ELOVL4 mutation in a case of spinocerebellar ataxia with erythrokeratodermia. JAMA Neurol..

[46] Sluch V.M., Banks A., Li H., Crowley M.A., Davis V., Xiang C., Yang J., Demirs J.T., Vrouvlianis J., Leehy B., Hanks S., Hyman A.M., Aranda J., Chang B., Bigelow C.E. (2018). ADIPOR1 is essential for vision and its RPE expression is lost in the Mfrprd6 mouse. Sci. Rep..

[47] Katoh M. (2001). Molecular cloning and characterization of MFRP, a novel gene encoding a membrane-type frizzled-related protein. Biochem. Biophys. Res. Commun..

[48] Kameya S., Hawes N.L., Chang B., Heckenlively J.R., Naggert J.K., Nishina P.M. (2002). Mfrp, a gene encoding a frizzled related protein, is mutated in the mouse retinal degeneration 6. Hum. Mol. Genet..

[49] Van Raay T.J., Vetter M.L. (2004). Wnt/frizzled signaling during vertebrate retinal development. Dev. Neurosci..

[50] Fogerty J., Besharse J.C. (2014). Subretinal infiltration of monocyte derived cells and complement misregulation in mice with AMD-like pathology. Adv. Exp. Med. Biol..

[51] Collery R.F., Volberding P.J., Bostrom J.R., Link B.A., Besharse J.C. (2016). Loss of Zebrafish Mfrp causes nanophthalmia, hyperopia, and accumulation of subretinal macrophages. Invest. Ophthalmol. Vis. Sci..

[52] Hawes N.L., Chang B., Hageman G.S., Nusinowitz S., Nishina P.M., Schneider B.S., Smith R.S., Roderick T.H., Davisson M.T., Heckenlively J.R. (2000). Retinal degeneration 6 (rd6): a new mouse model for human retinitis punctata albescens. Invest. Ophthalmol. Vis. Sci..

[53] Krill Alex E., Krill A.E., Archer D.B. (1977). Hereditary Retinal and Choroidal Dystrophies.

[54] Fogerty J., Besharse J.C. (2011). 174delG mutation in mouse MFRP causes photoreceptor degeneration and RPE atrophy. Invest. Ophthalmol. Vis. Sci..

[55] Velez G., Tsang S.H., Tsai Y.-T., Hsu C.-W., Gore A., Abdelhakim A.H., Mahajan M., Silverman R.H., Sparrow J.R., Bassuk A.G., Mahajan V.B. (2017). Gene therapy restores Mfrp and corrects axial eye length. Sci. Rep..

[56] Hankin J.A., Barkley R.M., Murphy R.C. (2007). Sublimation as a method of matrix application for mass spectrometric imaging. J. Am. Soc. Mass Spectrom..

[57] Kautzmann M.-A.I., Gordon W.C., Jun B., Do K.V., Matherne B.J., Fang Z., Bazan N.G. (2020). Membrane-type frizzled-related protein regulates lipidome and transcription for photoreceptor function. FASEB J..

[58] Zhang J., Wang C., Shen Y., Chen N., Wang L., Liang L., Guo T., Yin X., Ma Z., Zhang B., Yang L. (2016). A mutation in ADIPOR1 causes nonsyndromic autosomal dominant retinitis pigmentosa. Hum. Genet..

[59] Xu M., Eblimit A., Wang J., Li J., Wang F., Zhao L., Wang X., Xiao N., Li Y., Wong L.-J.C., Lewis R.A., Chen R. (2016). ADIPOR1 is mutated in syndromic retinitis pigmentosa. Hum. Mutat..

[60] Kaarniranta K., Paananen J., Nevalainen T., Sorri I., Seitsonen S., Immonen I., Salminen A., Pulkkinen L., Uusitupa M. (2012). Adiponectin receptor 1 gene (ADIPOR1) variant is associated with advanced age-related macular degeneration in Finnish population. Neurosci. Lett..

[61] Hollyfield J.G., Bonilha V.L., Rayborn M.E., Yang X., Shadrach K.G., Lu L., Ufret R.L., Salomon R.G., Perez V.L. (2008). Oxidative damage-induced inflammation initiates age-related macular degeneration. Nat. Med..

[62] Do K.V., Kautzmann M.-A.I., Jun B., Gordon W.C., Nshimiyimana R., Yang R., Petasis N.A., Bazan N.G. (2019). Elovanoids counteract oligomeric β-amyloid-induced gene expression and protect photoreceptors. PNAS.

[63] Gordon W.C., Bazan N.G. (1990). Docosahexaenoic acid utilization during rod photoreceptor cell renewal. J. Neurosci..

[64] Gordon W.C., Rodriguez de Turco E.B., Bazan N.G. (1992). Retinal pigment epithelial cells play a central role in the conservation of docosahexaenoic acid by photoreceptor cells after shedding and phagocytosis. Curr. Eye Res..

[65] Gordon W.C., Bazan N.G. (1993). Visualization of [3H]docosahexaenoic acid trafficking through photoreceptors and retinal pigment epithelium by electron microscopic autoradiography. Invest. Ophthalmol. Vis. Sci..

[66] Aveldaño M.I. (1988). Phospholipid species containing long and very long polyenoic fatty acids remain with rhodopsin after hexane extraction of photoreceptor membranes. Biochemistry.

[67] Aveldaño M.I., Sprecher H. (1987). Very long chain (C24 to C36) polyenoic fatty acids of the n-3 and n-6 series in dipolyunsaturated phosphatidylcholines from bovine retina. J. Biol. Chem..

[68] Aveldaño M.I., Bazán N.G. (1983). Molecular species of phosphatidylcholine, -ethanolamine, -serine, and -inositol in microsomal and photoreceptor membranes of bovine retina. J. Lipid Res..

[69] Aveldaño M.I., Pasquare de Garcia S.J., Bazán N.G. (1983). Biosynthesis of molecular species of inositol, choline, serine, and ethanolamine glycerophospholipids in the bovine retina. J. Lipid Res..

[70] Neuringer M., Connor W.E., Van Petten C., Barstad L. (1984). Dietary omega-3 fatty acid deficiency and visual loss in infant rhesus monkeys. J. Clin. Invest..

[71] Neuringer M., Reisbick S., Janowsky J. (1994). The role of n-3 fatty acids in visual and cognitive development: current evidence and methods of assessment. J. Pediatr..

[72] Hollyfield J.G. (2010). Age-related macular degeneration: the molecular link between oxidative damage, tissue-specific inflammation and outer retinal disease: the Proctor lecture. Invest. Ophthalmol. Vis. Sci..

